# The effect of COVID-19 on maternal newborn and child health (MNCH) services in Bangladesh, Nigeria and South Africa: call for a contextualised pandemic response in LMICs

**DOI:** 10.1186/s12939-021-01414-5

**Published:** 2021-03-15

**Authors:** Tanvir Ahmed, Ahmed Ehsanur Rahman, Taiwo Gboluwaga Amole, Hadiza Galadanci, Mushi Matjila, Priya Soma-Pillay, Bronwen M. Gillespie, Shams El Arifeen, Dilly O. C. Anumba

**Affiliations:** 1 Department of Oncology and Metabolism, Faculty of Medicine, Dentistry and Health, University of Sheffield, Sheffield, UK; 2 Maternal and Child Health Division, International Centre for Diarrhoeal Disease Research, Bangladesh (icddr,b), Dhaka, Bangladesh; 3 Department of Community Medicine and Africa Center of Excellence for Population Health and Policy (ACEPHAP), Bayero University, Kano, Nigeria; 4 Department of Obstetrics and Gynaecology Maternity Centre, Groote Schuur Hospital, Faculty of Health Sciences, University of Cape Town, Cape Town, South Africa; 5 University of Pretoria and Steve Biko Academic Hospital, Pretoria, South Africa

**Keywords:** COVID-19, MNCH, LMICs, Basic healthcare

## Abstract

Global response to COVID-19 pandemic has inadvertently undermined the achievement of existing public health priorities and laregely overlooked local context. Recent evidence suggests that this will cause additional maternal and childhood mortality and morbidity especially in low- and middle-income countries (LMICs). Here we have explored the contextual factors influencing maternal, neonatal and children health (MNCH) care in Bangladesh, Nigeria and South Africa amidst the pandemic. Our findings suggest that between March and May 2020, there was a reduction in utilisation of basic essential MNCH services such as antenatal care, family planning and immunization due to: a) the implementation of lockdown which triggered fear of contracting the COVID-19 and deterred people from accessing basic MNCH care, and b) a shift of focus towards pandemic, causing the detriment to other health services, and c) resource constraints. Taken together these issues have resulted in compromised provision of basic general healthcare. Given the likelihood of recurrent waves of the pandemic globally, COVID-19 mitigation plans therefore should be integrated with standard care provision to enhance system resilience to cope with all health needs. This commentary suggests a four-point contextualised mitigation plan to safeguard MNCH care during the pandemic using the observed countries as exemplars for LMIC health system adaptations to maintain the trajectory of progress regarding sustainable development goals (SDGs).

## Background

There is hardly any country, region or territory left in the world which has not yet been affected by the coronavirus pandemic. The year 2021 started with the grim global reality of roughly 87 million coronavirus cases and nearly two million related deaths. Due to higher proportions of reported cases, High-Income Countries (HICs) appear to have higher disease burdens. However, when ‘context’ is considered with ‘incidence and mortality rates,’ Low- and Middle- Income Countries (LMICs) appears to bear higher burden of COVID-19. For example, considering age and income, the HIC share of global mortality due to COVID-19 drops by a factor of 2.6 (from 78.9 to 30.7%) and rises three-fold for LMICs (from 21.1 to 69.3%) [1].

Irrespective of the disparity in impact, the global response to the COVID-19 pandemic has largely been a ‘one size fits all,’ centred around extensive lockdowns to ensure physical distancing whilst trying to maintain essential healthcare as much as possible [2]. Such approach has resulted in a shift of focus from essential healthcare services to providing mainly emergency services alongside COVID-19 care. In the past, the Ebola Virus infection resulted in additional health-related burden to the vulnerable groups such as mothers and children, the chronically ill (diabetic care, HIV/AIDS treatment), the elderly and people with disabilities including mental health challenges due to a decrease in the provision and utilization of usual healthcare [3]. Considering the fact that women and children are often the victims of poverty, ill-health and disparity, we have chosen to explore selected aspects of maternal, neonatal and child health (MNCH) care, to understand the effect of COVID-19 response on vulnerable groups in the context of LMICs.

Evidence from four LMICs with poor MNCH indices suggests that current coronavirus pandemic focused approach could lead to more than 30% additional maternal and newborn deaths due to reduced access to relevant essential services such as family planning, antenatal care (ANC) and adequately supervised community and facility-based deliveries [4]. Another study based on data from 118 LMICs estimated that the disruption in utilisation of MNHC services from the pandemic will increase under-5 mortality by 9·8–44·7% and maternal mortality by 8·3–38·6% per month, depending on the degree of disruption [5].

From the equity standpoint, conceptual frameworks like social determinants of health (SDH)^1^ or the work of the Social Exclusion Knowledge Network (SEKN)^2^ shows that social exclusion is a process which starts by undermining related political, economic and cultural factors [6]. These factors are key to the health of the vulnerable and socially excluded groups and are often neglected in blanketed or top-down approaches. As a result, vulnerable groups suffer the most and may become excluded further. Additionally, current pandemic focused approach is likely to cause additional public health crisis for mothers and children especially for the LMICs by disrupting access to usual healthcare and adding additional MNCH-related mortality and morbidity. Past Ebola experience suggests that it is of paramount importance for the LMICs to embark on a resilient health system which is adaptive enough to adopt approach to meet pandemic related challenges as well as continue to maintain focus on the pre-COVID healthcare needs/priorities. To do so, the first step is to explore, understand and contextualise how the pandemic has tested health system resilience [7]. Here, we have considered the disruption in utilisation of MNCH care as a result of the COVID-19 pandemic in three LMICs and highlight the need for a responsive health system approach to mitigate ongoing and future crises in MNCH care in these and other LMICs.

## Main text

Using the National Health Management Information System (HMIS) of Bangladesh and service data from two teaching hospitals both in Nigeria^3^ and South Africa^4^ where the HMIS was not readily available, we collected and compared information on utilization of selected MNCH services for 2 months during the pandemic (April/May 2020) and the same months in 2019. The services were selected from the list of SDG indicators and were operationalised into two groups: a) basic MNCH care that can be provided in the community or in the outpatient clinics of healthcare facilities (such as ANC, family planning (FP) and child immunisation services), and b) advanced MNCH care usually provided for patients admitted into healthcare facilities (such as normal vaginal deliveries (NVD) and caesarean sections (CS)). We then explored the underlying factors influencing the utilization of these MNCH services during the COVID-19 pandemic through informal discussion with key healthcare professionals and focused desk reviews of published scientific, grey and media-based information and country-specific healthcare policies. The findings were grouped and summarised by themes and are presented below.

### Utilisation of basic MNCH care has decreased during the COVID-19 pandemic

Table 1 shows that all three countries recorded a decline in attendance for formal ANC during April and May 2020 in comparison to the same months in 2019. Similarly, attendance at family planning clinics and for child immunisation declined in the countries where such data was available. There was a decline in facility vaginal deliveries in Bangladesh attributable to more homebirths, whilst the data appears more mixed for Nigeria and South Africa. It was partly due to limited data restricted to tertiary government facilities in both the african countries (Table 2). It is likely that the overall changes in deliveries in Nigeria and South Africa might have been influenced by resort to care in private facilities and tertiary facilities and home confinements during the pandemic. The more comprehensive data available for Bangladesh also shows a reduction in CS delivery rates, whilst the other two countries recorded a more mixed picture because the data obtained was limited to the few facilities studied.

**Table 1:** Utilisation (%) of basic MNCH care by months between 2019 and 2020

Country	Bangladesh	Nigeria		South Africa	
Indicator		AKTH	AWTH	GSH	SBAH
Mothers received ANC	March: ↓28.4% April: ↓56.9%	March: Suspended April: ↓65% May: ↓100%	March: ↓21% April: ↓85% May: ↓100%	April: ↓7.5%	April: ↓28.6%
Attendance in FP clinics	March: ↓100% April: ↓100%	April: ↓50% May: ↓72%	NA^b^	NA	NA
Children Vaccinated	^a^March: ↓13.5% April: ↓50.4%	April: ↓50%	NA	NA	NA

^a^Only Measles and Rubella Vaccine; ^b^*NA* Not Available

**Table 2:** Utilisation (%) of facility based MNCH care by months between 2019 and 2020

Country	Bangladesh	Nigeria		South Africa	
Indicator		AKTH	AWTH	GSH	SBAH
NVD	March: ↓31.7% April: ↓57.6%	April: ↓11.3% May: ↑9%	March: ↑24.5 April: ↑25.1 May: ↓7.3	March: ↓11% April: ↑14.3% May: ↑13.2%	March: ↑26.5 April: ↑39.6
CS	March: ↓50% April: ↓76.6.%	April: ↓10% May: ↑3.7%	March: ↓14.7 April: ↓18.8 May: ↓31.0	March: ↑4% April: ↑22% May: ↓2%	March: ↑7.2 April: ↑40.9
Total Deliveries (NVD + CS)	March: ↓40% April: ↓67%	April: ↓10.8% May: ↓5.4%	March: ↑14.1 April: ↑13.4 May: ↓17.1	March: ↓2% April: ↑19% May: ↑5%	March: ↑15.3 April: ↑40.4

### Factors associated with the decline in utilisation of basic MNCH care during the COVID-19 pandemic

Table 3 summarises when the first confirmed COVID-19 cases were reported in the three countries and outlines the measures for enforcing lockdowns and ensuring healthcare provision in these countries. After reviewing media and government reports, policy papers and scientific publications, we identified two main factors that caused the decline in utilisation of MNCH services in relation to the COVID-19 response: a) disruption of peoples’ lives due to lockdown and related measures and b) lack of safety measures for healthcare workers.

**Table 3:** Major timeline and related health system response in Bangladesh, Nigeria and South Africa

Traits	Bangladesh	Nigeria	South Africa
First confirmed case	March 08^a^	February 27^b^	March 05^d^
Beginning of lockdown	March 23^a^	March 24^c^	March 26^e^
Nature of lockdown	Initially for 10 days, later extended	State by state, partial	Nationwide
Health system response:	● Social distancing and provision of high-risk screening, hospitalization etc. and continuation of essential healthcare ● Bangladesh and South Africa converted specific tertiary hospitals to dedicated COVID-19 facilities; Nigeria made provision for COVID-19 care within existing facilities ● There was no specific MNCH care provision guideline in any of the countries. ● Electronic and print media-based information to raise awareness of benefits of social and interpersonal distancing, appropriate use of face masks and use of hand sanitizers, etc. ● Provision for periodic briefing updates on the pandemic by relevant government agencies/personnel.		

^a^World Health Organization (WHO) Bangladesh

^b^Nigeria Centre for Disease Control (NCDC)

^c^The Nation (www.thenationonlineng.net); March 24, 2020

^d^National Institute for Communicable Diseases, Govt. of South Africa (www.nicd.ac.za)

^e^Businesstech (www.businesstech.co.za); March 23, 2020

- *Lockdown regulations and the need for social distancing discouraged attendance in healthcare facilities including MNCH services, partly attributable to the fear of contracting the infection.*

The main mandate of enforcing the lockdown was to make people stay at home. This resulted in loss of income and reduced life-related activities. Usual health care seeking practices were severely reduced and mostly restricted to emergency healthcare needs. Our review of materials related to the pandemic response in all three countries did not reveal the provision of social distancing markings or signs at public places (e.g., marketplaces or bus stops) to help people maintain at least one-meter distance between two individuals. Many were unable to follow the norms of social distancing as they felt compelled to pursue earnings and societal interactions [8–10]. The lack of preparedness of countries in respect to the scale of the pandemic meant that no economic relief plan was put in place for the period of the lockdown. There did not seem to be sufficient financial plans to mitigate loss of earnings and the discomfort associated with social distancing. Although both Bangladesh and South Africa eventually announced a social relief and economic stimulus package, their impact on preventing or mitigating impending economic catastrophe across various socioeconomic groups is yet to be assessed.

b. *Lack of logistical support for healthcare providers and inadequate screening facilities made the circumstances unsafe for service provision.*

Globally, two of the most crucial components of COVID-19 guidance are the provision of screening facilities and the availability of personal protective equipment (PPE) and vaccines (when available) for healthcare staff. Consistent with reports elsewhere, our study countries experienced shortages is this area. Furthermore, people experiencing symptoms of COVID-19 often had to travel to remote designated facilities to provide samples which were then sent for testing at one of the very few designated screening facilities supporting the entire country. Results from such tests often took more than 1 week and such screening was deemed a precondition for gaining access to health facilities. People with medical emergencies often felt compelled to attend hospitals without test results. Some institutions were reported to have produced counterfeit screening test results, further compounding the risk of spread of COVID-19. Rationing of limited PPE and quarantine regulations for healthcare workers deemed to have exposed the healthcare providers to the infection and worsened healthcare provision further [10, 11].

The findings here show that the use of lockdown and social distancing measures as the universal COVID-19 response has undermined inherent community socio-economic dynamics by ignoring the social, political, economic and cultural (SPEC) factors, especially for the socially vulnerable groups. While such approach has affected health care priorities in LMICs, over time it is likely to cause the vulnerable groups to remain excluded from healthcare leading to the disparities in LMICs growing more.

It is important to note that, while the disruption pattern and factors have been similar to the HICs to some extent, there is emerging evidence that LMICs face higher mitigation challenges (factor of 30.7, 95% UI^5^ 14.7–48.8) compared to HICs (7.8, 95% UI 3.6–13.0) [12], a ncontext which have more robust, better financed and resilient health systems. On the other hand, such disruption in access will also affect other domains of healthcare. Hence, as projected by Roberton et al. [5], observations reported in this article are likely to be applicable to the increase mortality and morbidity of women and children and by extension to other vulnerable groups - chronically ill (diabetic care, HIV/AIDS treatment), elderly, people with disabilities including mental health challenges. However, on a global scale, the state of MNCH is a reference point for public health. Thus, the blanket coronavirus pandemic response is likely to undermine progress towards country-defined SDG targets and cause additional public health crises especially in the LMICs. To avoid this, innovative strategies in LMICs contexts should prioritise maintaining existing health priorities (e.g., MNCH) while responding to the challenges of the COVID-19 pandemic by adopting a holistic approach.

A recent commentary defines such a comprehensive approach as: “...a public health response that generates communication, understanding, learning, capabilities, civil responsibility, local innovations and global solidarity [13].” It is very encouraging that the WHO has recognised the importance of a comprehensive approach to the pandemic based on similar findings. An unsystematic search through  Google has shown about 200 reports, guidelines and checklists prepared by WHO relevant to the COVID-19 pandemic. Many of these documents have outlined a number of health system preparedness and response guidelines for the non-COVID services all of which are pertinent to women, children and adolescents’ healthcare. In addition, a handful of literature has suggested allocating additional resources, seeking local solutions, partnering with key public health programs and the use of technology such as telehealth. However, it is our understanding that for policy makers and other relevant stakeholders, it is very difficult to identify the starting point. This is now even more important given the present challenges of production and distribution of effective vaccines. While there is no simple solution, we think that the pandemic mitigation response must consider social, political, economic and cultural (SPEC) implications and address both COVID-19 and other non-COVID-19 health needs. Even with effective vaccine(s), there will be need for a) continued social distancing, personal hygiene and behaviour change to interrupt virus transmission and b) restoration of access to basic healthcare with additional effort to make up for the disruption in SDG. This will ensure restoration of normal life and socioeconomic activities. To embark on such a resilient health system response, with regards to MNCH services in LMIC contexts, the following measures would seem appropriate starting points:

1. Local MNCH care providers and managers need to be consulted to understand the breadth of the socio-economic impact of COVID-19 and COVID-19 response measures, and their relation to MNCH care provision. This can be an opportunity to consider locally acceptable measures to improve compliance with social distancing and identifying the needs of the local healthcare providers.
2. The COVID-19 mitigation plans need to be segregated by the tiers of the health system (e.g. primary, secondary and tertiary) of the respective country. Such operationalisation will help in identifying the scope of MNCH care providers and managers at different levels to help adapt the COVID-19 response to the specific context.
3. An efficient and robust combination of community-based education and COVID-19 testing with essential training can promote the continuing provision of existing MNCH services amidst COVID like pandemics whilst ensuring appropriate essential task shifting and limiting duplication and wastage of resources.
4. The COVID-19 mitigation strategies should be integrated and embedded within the existing HMIS of the respective countries, in order to facilitate acquisition of data on trends, thereby helping to generate evidence-based policy decisions to inform resource allocation and tracking of MNCH and other non-COVID-19 services as well as COVID-19 services.

## Conclusions

Considering the inevitability of multiple waves of the COVID-19 pandemic globally, consideration of political, economic and contextual factors in formulating appropriate responses is crucial for a resilient health system. Communities and health professionals can help inform locally designed approaches to ensure more effective non-draconian social distancing, use of masks, and adoption of effective vaccines when the latter becomes available. The key should include coordination between actors through more efficient use of various approaches, including digital platforms, to establish communication and information and reporting channels. In addition, further research is required in LMIC contexts to enable culturally relevant and context-appropriate approaches to address the health care challenges posed by this and future pandemic(s) whilst maintaining other essential health care services including MNCH.

## Data Availability

The datasets used and/or analysed during the current study are available from the corresponding author on reasonable request.

## Abbreviations

COVID-19: Coronavirus Disease 2019
LMICs: Low- and Middle-Income Countries
MNCH: Maternal, Neonatal and Children Health
SDGs: Sustainable Development Goals
HICs: High-Income Countries
ANC: Antenatal Care
HMIS: Health Management Information System
FP: Family Planning
NVD: Normal Vaginal Deliveries
CS: Caesarean Section
WHO: World Health Organization
PPE: Personal Protective Equipment

## Footnotes

1 Social determinants of Health (SDH) shows how non-medical factors determines health outcome and thereby are crucial to understand health related disparity in a population (https://www.who.int/health-topics/social-determinants-of-health#tab=tab_1).

2 “Social Exclusion Knowledge Network (SEKN) focus on and examine the relational processes that work to exclude particular groups of people in particular contexts from engaging fully in community/social life” (https://www.who.int/social_determinants/resources/sekn_scoping.pdf).

3 Nigeria - Aminu Kano Teaching Hospital (AKTH) and Abdullahi Wase Teaching Hospital (AWTH), Kano

4 South Africa: Groote Schuur Hospital (GSH), Cape Town and Steve Biko Academic Hospital, Pretoria

5 UI refers to Uncertainty Intervals, a philosophically appropriate synonym of Confidence Interval which refers to the degree of uncertainty of the corresponding statistics.
